# Effect of domestication on the genetic diversity and structure of *Saccharina japonica* populations in China

**DOI:** 10.1038/srep42158

**Published:** 2017-02-08

**Authors:** Jie Zhang, Xiuliang Wang, Jianting Yao, Qiuying Li, Fuli Liu, Norishige Yotsukura, Tatiana N. Krupnova, Delin Duan

**Affiliations:** 1 Key Lab of Experimental Marine Biology, Institute of Oceanology, Chinese Academy of Sciences, Qingdao 266071, China; 2 Laboratory for Marine Biology and Biotechnology, Qingdao National Laboratory for Marine Science and Technology, Qingdao 266071, China; 3 University of Chinese Academy of Sciences, Beijing, 100049, China; 4 Yellow Sea Fisheries Research Institute, Chinese Academy of Fishery Sciences, Qingdao, 266071, China; 5 Field Science Centre for Northern Biosphere, Hokkaido University, Sapporo, 060-0809, Japan; 6 Pacific Research Fisheries Centre (TINRO-Centre), Vladivostok, 690600, Russia

## Abstract

*Saccharina japonica* is a commercially and ecologically important seaweed and is an excellent system for understanding the effects of domestication on marine crops. In this study, we used 19 selected simple sequence repeat (SSR) markers to investigate the influence of domestication on the genetic diversity and structure of *S. japonica* populations. Wild kelp populations exhibited higher genetic diversity than cultivated populations based on total *N*_A_, *H*_E_, *H*_O_, *N*_P_ and *A*_R_. Discriminant analysis of principal components (DAPC), a neighbour-joining (NJ) tree and STRUCTURE analyses indicated that *S. japonica* populations could be divided into two groups (a cultivated/introduced group and a wild indigenous group) with significant genetic differentiation (*P *<* *0.0001). Divergent selection, continuous inbreeding and inter-specific hybridization have caused the divergence of these two genetically separate gene pools. The significant genetic differentiation between northern and southern cultivated populations appears to be due to inter-specific hybridization and wild germplasm introduction during the domestication process. In addition, the cultivation of *S. japonica* has not resulted in any serious genetic disturbance of wild introduced *S. japonica* populations. An understanding of the genetic diversity and genetic structure of domesticated *S. japonica* will be necessary for further genetic improvement and effective use of germplasm.

Approximately 13,000 years ago, humans began to domesticate plants and animals^1^. Worldwide cultivation of seaweeds, however, has a much shorter history, increasing substantially over the last 50 years^2^. Seaweed aquaculture has been developed primarily in Asia^2^,^3^, and to date, fewer than 20 species have been domesticated^4^. More than 90% of worldwide farming activities concentrate on five taxa^2^,^5^,^6^: the brown kelps *Saccharina* and *Undaria* and the red algae *Porphyra, Gracilaria* and *Eucheuma.*

Plant domestication not only modifies economic and agronomic phenotypes but also leaves a genetic signature that affects both the population structure and the genetic diversity of the domesticated species^7^,^8^. For marine crops, previous studied indicated that an extended period of inbreeding and intensive selection during the domestication process may reduce the genetic diversity, narrow the germplasm base and promote adaptive divergence between the domesticated seaweeds and their wild counterparts^8^,^9^,^10^,^11^. An understanding of how domestication affects the domesticated species provides insights into general mechanisms of adaptions and diversification and can guide the genetic improvement of crops in breeding programs^1^.

*Saccharina japonica* (Aresch) Lane, Mayes, Druehl & Saunders is native to cold-temperature coasts along northern Japan, northwestern Korea and Far Eastern Russia and was introduced into China in the 1930s^12^. *S. japonica* is a commercially and ecologically important seaweed that is mainly cultivated in China, Japan and Korea. China, in particular, ranks first in the world for the cultivation of this species with a wet weight production of 5,941,658 t in 2013 (FAO: http://www.fao.org/fishery/statistics/global-aquaculture-production). *S. japonica* was initially cultivated in the north of China using conventional methods (e.g., tying sporelings to rocks) as early as the 1930 s, but modern cultivation started in the 1950s^12^,^13^ and is successfully conducted in China on a large scale today (Supplementary Fig. S1). The expansion of the kelp cultivation industry in China can mainly be attributed to the development and implementation of the summer sporelings method^13^,^14^,^15^, the floating raft cultivation technique and fertilizer application^13^,^15^,^16^,^17^. To expand the cultivation area, a considerable effort was made to transplant kelp from the northern areas (Shandong and Liaoning, 35–39°N in latitude) to the south of China (Zhejiang and Fujian, 24–31°N in latitude)^13^,^14^,^18^.

Early in the domestication process, wild *S. japonica* populations from Japan or wild Chinese introduced populations were cultivated rather than selected strains^14^. Since the 1960 s, selective breeding has been applied to *S. japonica*, the first variety to be produced being “Haiqing No. 1”^19^. Subsequently, various kelp breeding strategies including gametophyte cloning, intra- and inter-specific hybridization methods and heterosis have been used to produce elite northern cultivars with high yield and improved resistant to light irradiance such as 901, Rongfu, Dongfang No. 2 and Dongfang No. 3^14^,^20^,^21^,^22^,^23^. Most southern cultivars have been bred using continuous selection strategies based on local cultivar germplasm, such as Huangguan No. 1^24^.

There is evidence that domestication has led to a reduction in genetic diversity for several cultivated seaweeds, including *Undaria pinnatifida, Ulva prolifera* and *Porphyra yezoensis*^9^,^10^,^11^. Some previous reports have assessed the genetic diversity and population structure of *S. japonica* populations in China and indicated that extensive selection and inbreeding for multiple generations are likely to also have reduced the genetic diversity of cultivated *S. japonica* populations^25^,^26^,^27^,^28^. Both kelp germplasm introduction and inter-specific hybridization were expected to increase the genetic diversity by introducing the new genetic variations into the cultivated strains. However, little is known about the effects of these two processes on the genetic structure of the populations of *S. japonica* that are cultivated at present. In addition, some studies showed that the escaped cultivated kelp from the farms might cross-hybridize with wild populations, resulting in genetic introgression of cultivar genetic material into wild populations^3^,^5^,^11^,^29^. It is not known whether a similar gene flow from cultivated to wild populations has occurred for *S. japonica*. Estimating the existence and extent of such a gene flow might be helpful for designing breeding practices and conservation strategies.

Using selected microsatellite markers, we conducted a population genetic analysis of 28 *S. japonica* populations (17 cultivated populations, 3 wild introduced populations and 8 wild indigenous populations). The objectives of our study were 1) to examine if Chinese populations after domestication are less genetically diverse than wild indigenous populations, a pattern expected given the known history of the introduction of *S. japonica* in China, 2) to test the prediction that kelp germplasm introduction and inter-specific hybridization will increase the genetic diversity and enlarge the genetic divergence of cultivated *S. japonica* populations by introducing the new genetic variations, and 3) to evaluate the genetic diversity of wild introduced populations and investigate whether escapes from cultivated populations influence the wild introduced populations. The long-term objective is to improve selection and breeding and sustainable utilization of *S. japonica* seed stocks.

## Results

### Selection and characteristics of the SSR loci

**Figure 1:**
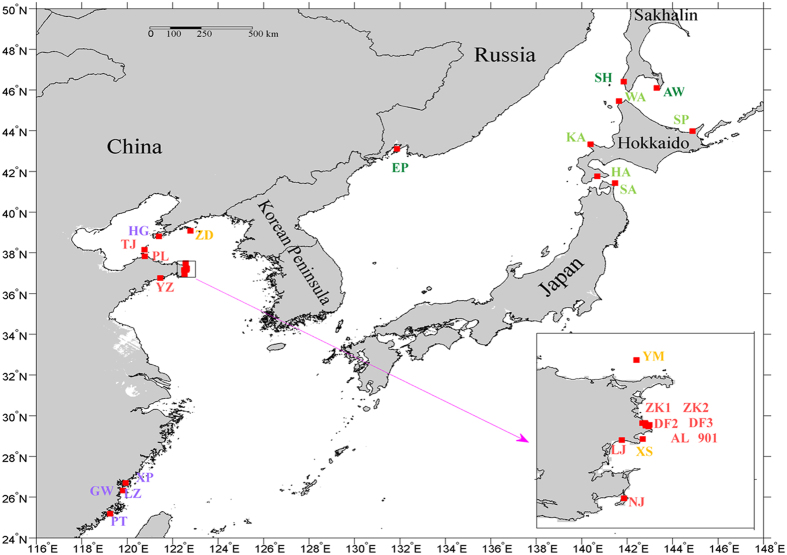
Geographic locations of the domesticated and wild *Saccharina japonica* populations used in this study. The geographic figure was created using the MATLAB software package (R2012b) (http://cn.mathworks.com/products/matlab/). Red indicates northern cultivated populations; Purple shows southern cultivated populations; Orange indicates wild introduced populations; Dark green shows Russian wild populations; Light green represents Japanese wild populations.

A total of 766 individuals from 28 populations (Fig. 1; Supplementary Table S1) were genotyped at 23 nuclear SSRs derived from genomic data^30^. The percentage of missing genotypes from the whole dataset was 0.085% (30 missing genotypes for 35,236 attempted). The percentage of successful amplifications per locus ranged from 96% to 100%. The number of alleles (*A*) ranged between 7 for SJ93 and 50 for SJ136 (Supplementary Table S2). The SSR markers exhibited polymorphism with high polymorphic information content (PIC) (PIC > 0.5) for 22 SSR markers and low PIC (0.22) for SJ31 (Supplementary Table S2). The mean expected heterozygosity across populations ranged from 0.18 ± 0.05 to 0.72 ± 0.04, and the mean observed heterozygosity ranged from 0.16 ± 0.04 to 0.68 ± 0.04. microchecker detected no genotyping error due to stuttering and large allele dropout, but null alleles were detected at several loci: SJ13, SJ21, SJ125 and SJ136. We used the software program freena to estimate the average frequency of null alleles per locus, and this varied from 0.00 ± 0.00 for SJ3 to 0.16 ± 0.02 for SJ136. Only four loci (SJ13, SJ21, SJ125 and SJ136) had high frequencies of null alleles (>0.06) (Supplementary Table S2). The global *F*_ST_ across all loci without correction for null alleles (0.342, 95% CI: 0.307–0.378) was slightly higher than the corrected *F*_ST_ values (0.333, 95% CI: 0.299–0.368), and the pairwise *F*_ST_ per locus without correction was also higher than the pairwise *F*_ST_ with correction (data not shown). High frequencies of null alleles have the potential to influence the estimation of genetic differentiation. Consequently, we excluded these four loci (SJ13, SJ21, SJ125 and SJ136) from this study and reported only the results based on 19 SSRs.

After false discovery rate (FDR) correction for multiple tests, linkage disequilibrium (LD) tests for each pair of loci indicated that 171 pairs (3.5%) were significantly in disequilibrium. Given that these loci did not share corresponding disequilibria in all samples, we assumed that none of the loci were physically linked. No consistent pattern of linkage disequilibrium was observed, so the 19 loci were used for all subsequent analyses.

### Summary statistics for 28 *S. japonica* populations

Genetic diversity was evaluated for 28 *S. japonica* populations at the population and group levels (Table 1). At the population level, the mean number of alleles across loci (*N*_A_) varied from 1.79 ± 0.10 for XP (one southern cultivated population from China) to 9.11 ± 1.24 for SA (one wild indigenous population from Shiriya, Aomori pref, Japan). Allelic richness (*A*_R_) based on 20 samples per population was highest (8.19 ± 1.07) in the SA population and lowest (1.78 ± 0.10) in the XP population. There were no private alleles in the cultivated populations, but private alleles existed in all wild introduced/indigenous populations (WI + WR + WJ) except one wild introduced population (YM). The mean observed heterozygosity across loci (*H*_O_) ranged from 0.25 ± 0.05 for XP to 0.68 ± 0.04 for HA (one wild indigenous population from Hakodate, Japan), and expected heterozygosity across loci (*H*_E_) ranged from 0.25 ± 0.04 for XP to 0.71 ± 0.04 for HA.

We compared the mean values of all genetic diversity indices (*N*_A_, *A*_R_, *N*_P_, *H*_O_ and *H*_E_) at the group level (Table 1) and found that all parameters were highest in the wild indigenous populations in Japan (WJ) and lowest in southern cultivated populations (SC). In addition, the genetic diversity of wild indigenous populations (WJ and WR, *H*_S_ = 0.539) was significantly higher (*P* = 0.003; Supplementary Table S3) than the genetic diversity of cultivated populations (NC and SC, *H*_S_ = 0.390). The genetic diversity indices in northern cultivated populations (NC, *H*_S_ = 0.415) were higher than in wild introduced populations (WI, *H*_S_ = 0.386) and southern cultivated populations (SC, *H*_S_ = 0.328) (Supplementary Table S3).

*F*_IS_ values showed significant deviation from zero (*P* < 0.01, Table 1) in the ten populations, indicating departures from Hardy–Weinberg equilibrium. Five cultivated populations (901, DF2, PL, HG and XP) had significantly negative *F*_IS_ values, indicating heterozygosity excess, whereas five wild populations (ZD, AW, KA, HA and SA) had significantly positive *F*_IS_ values, indicating heterozygote deficiency. After FDR correction, the DF2 population showed significant departures from HWE, involving 16 of the 19 loci, all due to heterozygote excess.

Recent changes in effective population size were detected based on the Wilcoxon signed rank-test under the infinite allele model (IAM), the stepwise-mutation model (SMM), and the two-phase mutation model (TPM). Eight cultivated populations and two wild introduced populations showed significant heterozygote excess compared to the expected equilibrium in the IAM after FDR correction (Supplementary Table S4). The TPM model has been reported to be the most conservative and powerful model^31^, and the results obtained with the TPM model indicated that the cultivated population DF2 showed significant heterozygosity excess compared to the expected equilibrium after FDR correction (Supplementary Table S4). However, hybridization can severely influence the outcome of the bottleneck tests, so the significant heterozygosity excess of the DF2 population is possibly due to hybridization during the breeding of this variety. The mode-shift test detected characteristic mode-shift distortion in the typical L-shape distribution of allele frequencies caused by bottlenecks in DF2 and XP.

### Two major genetic groups: a wild indigenous group and a cultivated/introduced group

**Figure 2:**
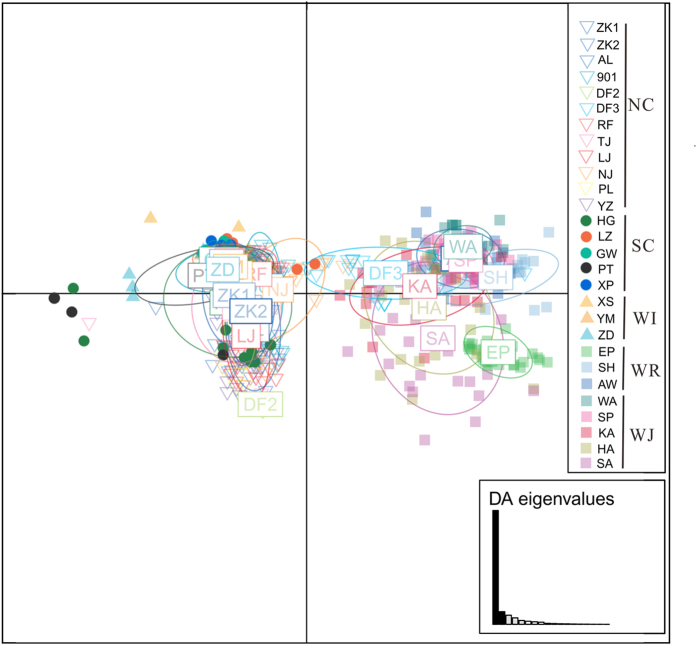
Scatterplot output from a discriminant analysis of principal components (DAPC) for the genetic structure of *Saccharina japonica* individuals based on 19 microsatellites. Dots represent individuals from the 28 populations, and different populations are depicted with different colours and symbols. Abbreviations correspond to populations presented in Table 1. The bar graph inset exhibits the variance explained by the two discriminant eigenvalues used for plotting. The 67% inertia ellipses are drawn for each population, representing the variance of the two principal components.

**Figure 3:**
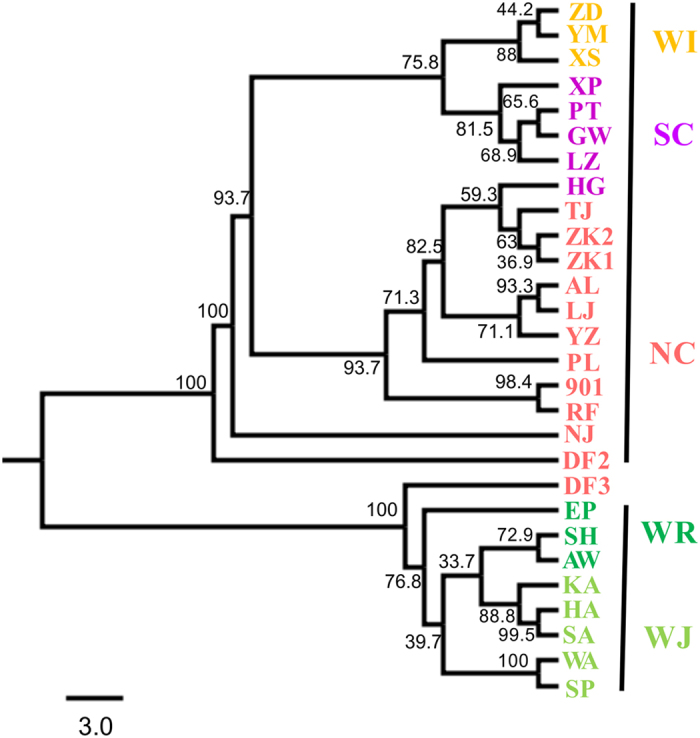
Neighbour-joining tree constructed based on Nei’s standardized genetic distance (*Da*) for 28 *Saccharina japonica* populations.

**Figure 4:**
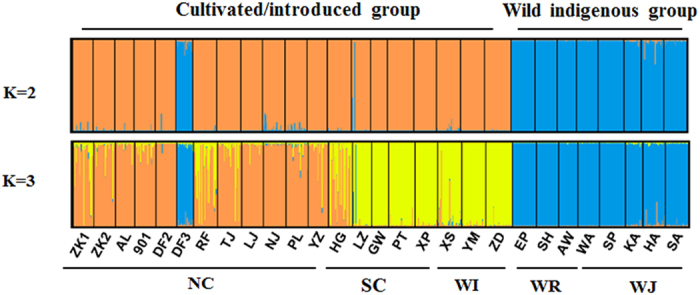
Bayesian estimates of population structure based on SSR data for the whole dataset. The results (K = 2 and K = 3) obtained in STRUCTURE are shown for comparison. The vertical bar indicate individuals, and colours correspond to specific clusters.

Discriminant analysis of principal components (DAPC) partitioned the *S. japonica* populations into two genetic clusters (Fig. 2). This partitioning was supported by the lowest Bayesian information criteria (BIC) in the DAPC analyses when K = 2. DAPC analyses revealed two genetic clusters: a wild indigenous cluster, including Russian and Japanese wild populations (WR + WJ), and a cultivated/introduced cluster, which included all populations from China (NC + SC + WI) except for DF3 (Fig. 2). A neighbour-joining (NJ) analysis calculated with Nei’s pairwise genetic distance (*Da*) indicated that the wild indigenous cluster of populations was clearly distinct from the cultivated/introduced cluster (except DF3) (Fig. 3). Bayesian clustering of the 28 populations was carried out with STRUCTURE, and consistent results were obtained from the 20 runs to test for each K value. The natural logarithm of the likelihood of the data increased sharply from K = 1 to K = 2 (Supplementary Fig. S2A). The metric delta K peaked at K = 2, indicating that this was the highest hierarchical level of the genetic structure (Supplementary Fig. S2B; delta K = 9903.50). Consistent with the NJ tree and DAPC results, the cultivated/introduced group and wild indigenous group (K = 2) could be distinguished in the STRUCTURE analysis and showed very little admixture between them (Fig. 4). A second hierarchical level of genetic division was identified with K = 3 (Supplementary Fig. S2B; delta K = 87.90), indicating that the cultivated/introduced group (except DF3) could be divided into two genetic subgroups: one containing the northern cultivated populations (NC) and the other the southern cultivated populations (SC) and the wild introduced populations (WI) (Fig. 4). Wild introduced populations (WI) and southern cultivated populations (SC) clustered together, and this was also supported by the NJ tree. The southern and northern cultivated kelp populations could be clearly separated, but three cultivated populations (ZK1, RF, HG) had high genetic admixture (Fig. 4). Most of the populations clustered according to geographical distribution in DAPC plots, NJ tree and the STRUCTURE analysis, except for the DF3 population. From the NJ tree, seven cultivated populations (TJ, ZK1, ZK2, AL, LJ, YZ and PL) bred from intra-specific crosses clustered together and were genetically separated from other cultivated populations (901, RF, DF2 and DF3) derived from inter-specific crosses (Fig. 3).

### Patterns of genetic differentiation

Pairwise *F*_ST_ values with and without correction for null alleles showed some slight variation with differences ranging from 0.000 to 0.026 (data not shown). Of the 378 pairwise tests conducted among 28 kelp populations, 370 were highly significant (*P* < 0.0001), and 6 were significant (*P* < 0.05) after FDR correction (Supplementary Table S5). Therefore, most of the 28 populations have significant genetic differentiation from each other. *F*_ST_ values ranged from 0.009 (ZK1 and ZK2) to 0.695 (XP and EP) (Supplementary Table S5). The Chinese populations (except DF2 and DF3) were genetically divergent from wild indigenous populations (*F*_ST_: 0.277–0.695; *P* < 0.0001). However, pairwise *F*_ST_ values between DF3 and three Japanese populations (KA, HA and SA) varied from 0.152 to 0.240, which indicated moderate genetic differentiation among those populations (Supplementary Table S5). There was not a high level of genetic differentiation among most of the cultivated/introduced populations (NC + SC + WI) (0.009–0.330), but DF2 and DF3 had higher pairwise *F*_ST_ values (0.224–0.486) compared with other cultivated populations. In addition, two northern cultivated populations (901 and NJ) were highly differentiated from three southern cultivated populations (GW, XP and PT) with *F*_ST_ values ranging from 0.252 to 0.330. When *F*_ST_ values among populations within each group were calculated independently, the highest value was found within the WR group (*F*_ST_ = 0.305) and the lowest value (*F*_ST_ = 0.063) within the WI group (Supplementary Table S3). In addition, the genetic differentiation between wild indigenous populations (WR + WJ, *F*_ST_ = 0.247) was significantly larger than for cultivated populations (NC + SC + WI, *F*_ST_ = 0.199) (Supplementary Table S3).

We detected significant genetic differentiation between wild indigenous and cultivated/introduced groups, and this separation was confirmed by an estimation of 30.49% genetic variance between these two groups based on AMOVA (*Ф*_CT_ = 0.30; *P* < 0.001) (Table 2). The genetic variance partitioned between populations within groups was 14.67% (*Ф*_SC_ = 0.21; *P* < 0.001), while 54.85% of the variation existed within populations. AMOVA results based on northern and southern cultivated populations showed significant *Ф-*statistics among populations (*Ф*_ST_ = 0.23; *P* < 0.001), among populations within groups (*Ф*_SC_ = 0.17; *P* < 0.001) and among groups (*Ф*_CT_ = 0.06; *P* < 0.001). All variance components were statistically significant (*P* < 0.05, Table 2).

## Discussion

Generally, domestication tends to reduce allelic variation and genetic diversity inducing genetic erosion in cultivated seaweeds^8^. This phenomenon has been observed for *U. pinnatifida, U. prolifera* and *P. yezoensis*^9^,^10^,^11^. In the present study, analysis of mean values of *N*_A_, *H*_E_, *H*_O_, *N*_P_, *A*_R_ and *H*_S_ in each group indicated that genetic diversity was higher in the Japanese wild populations than in the domesticated kelp populations. This difference may be due to continuous selection having reduced the effective population size and increased genetic drift and hitchhiking during the domestication process^8^,^32^,^33^. Continuous inbreeding and directional selection were usually adopted for the breeding of *S. japonica* in China^19^,^26^. Although there were no obvious signs of inbreeding in cultivated populations, we cannot exclude that inbreeding and selfing during the breeding process caused genetic erosion in the cultivated populations, as was reported in previous studies^26^,^27^,^34^.

Departures from Hardy–Weinberg expectations due to heterozygosity excess were detected in five *S. japonica* cultivated populations (901, DF2, PL, HG and XP) (Table 1), possibly due to a human-mediated non-random mating strategy (small reproductive population size, existence of heterosis and effect of gametophytic self-incompatible system). One possible explanation of heterozygote excess in these cultivated populations may be the small reproductive population size during the breeding process. When only a few parental kelp contribute to the next generation, allelic frequencies can differ between male and female parents, and lead to a significant deviation from random mating^35^,^36^. Another potential explanation was that selection for linked heterozygotes or associated heterosis effect could cause the heterozygote excess in these cultivated populations. To fully utilize heterosis to breed the seedlings for enhancing the quality and quantity of the next year’s cultivation, intraspecific hybridization of divergent lineages or interspecific hybridization could be used. Such an effect of heterosis is exemplified by the DF2 population: the cultivar Dongfang NO. 2 (DF2) was the first filial generation obtained by hybridizing a male gametophyte clone of *S. longissima* with a female one of *S. japonica*^20^, and in our study, it showed particularly significant heterozygosity excess (*F*_IS_ = 0.96; *P* < 0.01). Therefore, we presumed that overdominant selection in the breeding cultivars favoured heterozygote survival and caused the heterozygote excess with hitch-hiking selection. One common explanation for observing heterozygote excess is the self-incompatibility system effect^37^; however, we could not conclude that the excess of heterozygotes in cultivated *S. japonica* was caused by active avoidance of selfing and full-sibling mating.

In contrast to cultivated populations, five wild populations showed significant departure from HWE with heterozygote deficiencies. An excess of homozygosity within wild populations can be due to at least three causes: the presence of null alleles, inbreeding and population subdivision. Inbreeding could be common in wild populations, possibly due to the limited dispersal of the kelp gametes, favouring mating within the kelp siblings or close relatives^38^,^39^. Our present data are not sufficient to resolve whether there was a substructure within our populations that could result in a Wahlund effect^40^. However, in a previous study of local substructure and gene flow in wild *S. japonica* indigenous populations^41^, we found evidence to support a within-population structure as a cause of heterozygote deficiency.

In this study, we observed that wild introduced populations (WI) had lower genetic diversity than wild indigenous populations (WR + WJ). This was consistent with our previous results that wild introduced populations have lower haplotype and nucleotide diversity than wild indigenous populations^41^. The reduction of genetic diversity in wild introduced populations was possibly due to founder effects and genetic drift following introduction in the 1930 s. This is supported by the fact that two wild introduced populations (YM and ZD) showed signs of a genetic bottleneck (Supplementary Table S4). Moreover, some wild introduced populations were transplanted from Shandong (35–36°N) to Zhejiang (27–31°N) and to Fujian (24–27°N) in China in the 1960s^14^,^16^,^18^. The southward transplantation had a profound influence on the genetic structure of the cultivated *S. japonica* populations. DAPC plots, an NJ tree and STRUCTURE analyses indicated that the clustering of the southern cultivated populations (SC) and the wild introduced populations (WI) was principally driven by these transplantations (Supplementary Fig. S1).

The northern cultivated populations (NC) and the wild introduced populations (WI) all grow in the north of China, but they did not cluster together in our analyses. We believe that the northern cultivated populations (NC) are genetically different and diverse as a result of interspecific hybridization and/or multiple introductions of wild indigenous germplasm^12^,^20^,^21^,^23^,^42^, while the wild introduced populations (WI) are comparatively more isolated genetically, with less germplasm introduction. There was evidence for a low level of gene admixture between cultivated populations and one wild introduced population (XS) (8.4% wild introduced individuals belong to cultivated populations), while no evidence was found for gene admixture among the other wild introduced populations (YM and ZD) and the cultivated populations (Fig. 4). These results imply that the wild populations of *S. japonica* have not been markedly impacted by gene flow from cultivated populations.

Several earlier studies reported that northern and southern cultivated kelp populations lacked any significant genetic differentiation^26^,^27^. Although the degree of differentiation between northern and southern cultivated populations is globally mild (only 6% variance explained by northern *vs.* southern differences), our analyses indicated significant genetic differentiation among these cultivated populations (*Ф*_CT_ = 0.06; *P* < 0.001) (Table 2). The difference between the populations is principally due to the northern cultivated populations (NC) representing a genetically mixed gene pool with repeated introductions of wild indigenous germplasm (from the same species or from congeneric species). Southern cultivated populations (SC) seem to have retained the original genetic composition of the wild introduced populations (WI), with less germplasm introduction.

Most northern cultivated populations from inter-specific and intra-specific crosses have distinct genetic structure patterns. Indeed, most cultivated populations bred from descendants of *S. japonica* clustered together and were separated from descendants of inter-specific crosses in the NJ tree (Fig. 3). Liu *et al*. reported two cultivars (901 and DF3) that were derived from hybridization between *S. japonica* and *S. longissima* clustered together^26^. In our study, it was surprising that 901 clustered with RF, not DF3 (Fig. 3), although cultivar “Rongfu” (RF) contained wild germplasm from *S. latissima*^22^. We presumed that these two cultivars might have been genetically mixed in the farms, due to deliberate hybridization by farmers or uncontrolled genetic mixing of cultivars during the summer sporeling-rearing^27^.

Based on the STRUCTURE analyses and NJ tree, the northern cultivated population DF3 clustered with wild indigenous populations. We suspected that this cultivar might have been unconsciously or deliberately mixed with wild kelp germplasm by farmers during the breeding or cultivation processes. To verify whether there is contamination and degradation in these DF3 varieties, a detailed study of the DF3 strains cultivated in different farms should be further conducted.

In conclusion, the overall genetic structure of the *S. japonica* strains analysed in this study suggest that this cultivated kelp represents a single complex gene pool within which historical movement of germplasm, recent introductions, interspecific hybridizations and human selection are shaping the genetic structure. In our present study, the expected reduced diversity of cultivated populations was confirmed, and genetically distant populations or geographically isolated populations should be used for enhancing diversity and improving productivity. Diverse wild *S. japonica* resources are essential for the restoration and selection processes necessary for Chinese kelp production. Further genome-wide analysis of the domesticated kelps will provide more information to understand in detail the micro-evolution processes that have occurred during the domestication and thus to improve kelp breeding strategies.

## Methods

### Sample collection and identification

We sampled 28 *S. japonica* populations (766 individuals), including twelve northern cultivated populations (NC), five southern cultivated populations (SC), three wild introduced populations (WI) and eight wild indigenous populations from Russia and Japan (WR and WJ) (Supplementary Table S1; Fig. 1). Seventeen of these populations (458 individuals) had already been used in previous studies including seven cultivated populations^26^ (174 individuals) and ten wild introduced/indigenous populations^41^ (284 individuals) (Supplementary Table S1). Six of the cultivated cultivars have been examined and approved by the Chinese Approving Committee of Aquacultural Stock Seeds and Elite Varieties: “Huangguan No.1” (HG)^22^,^24^, “Rongfu” (RF)^17^, “Ailunwan” (AL), “90-1” (901)^23^,^34^, “Dongfang No.2” (DF2)^20^ and “Dongfang No.3” (DF3)^21^, and the remaining 11 cultivated populations corresponded to production cultivars that are commonly employed in kelp production. Wild introduced populations corresponded to populations from China that are able to complete their life histories in the wild, whereas the wild indigenous populations corresponded to Russian (3 populations) and Japanese populations (5 populations) that might have been the original source of strains cultivated in China.

### SSR analysis

Twenty-three simple sequence repeat (SSR) markers were screened from a list of 76 SSRs previously developed by Li *et al*.^30^ and analysed using three sets of multiplex PCR reactions in this study (Supplementary Table S6). Each multiplex set was carefully assembled based on the compatibility of SSRs during the PCR and on the molecular size of their amplicons. Forward primers of SSR markers were labelled with one of the following fluorescent dyes: carboxy fluorescein (FAM), carboxytetrame thylrhodamine (TAMRA) or hexachloro-6-carboxy fluorescein (HEX). The reaction mixtures (10 μL) contained 0.2 μL template DNA, 0.1 μL of each primer, and 5 μL Multiplex PCR Master Mix (QIAGEN Multiplex PCR Kit, Qiagen, Germany). The PCR reactions were performed in a Life Pro thermocycler with an initial denaturation step of 5 min at 95 °C, followed by 20 cycles of 95 °C for 30 s, 55–58 °C for 30 s and 72 °C for 30 s, and a final extension at 72 °C for 10 min. For PCR fragment size determinations, 0.5 μL of an internal size standard (Liz-500, LIZ) was mixed with 0.3 μL of PCR product and 9.5 μL formamide. The mixture was heated to 95 °C for 5 min, then cooled down on ice, and finally subjected to fragment analysis on an ABI PRISM 3730 sequencer (Applied Biosystems, Unites States of America). The alleles were sized using genemarker v2.2.0 (SoftGenetics).

### Statistical analysis

Micro-checker 2.2.3 was used to check for potential genotyping errors caused by stuttering or large allele dropout and the presence of null alleles^43^. Null allele frequencies across the populations were estimated using the expectation maximization (EM) algorithm by the program freena^44^. Linkage disequilibrium (LD) and deviations from Hardy–Weinberg equilibrium (HWE) were estimated for each SSR locus and individual kelp populations using genepop 4.2.2^45^ with 10,000 dememorization and in 20 batches with 5,000 iterations per batch. Multiple tests in the detection of LD and HWE were corrected using the false discovery approach^46^ in the R-package qvalue (R Development Core Team 2013). For polymorphism evaluation of each SSR locus, allele numbers (A), expected and observed heterozygosity (*H*_E_ and *H*_O_), and polymorphic information content (PIC) were calculated using popgene 1.3.1^47^. For measures of genetic diversity in each population, the mean number of alleles across all loci (*N*_A_) and allelic richness (*A*_R_) across all loci based on the minimal sample size with the rarefaction method were calculated in fstat 2.9.3.2^48^. The mean observed and expected heterozygosity (*H*_E_ and *H*_O_) and numbers of private alleles (*N*_P_) were calculated using genalex 6.41^49^. The significance of departures from Hardy–Weinberg equilibrium, as evidenced by deviation of inbreeding coefficient (*F*_IS_) from zero, was tested with 5,000 randomizations using fstat 2.9.3.2^50^. Microsatellite data files were converted into the formats for the various analysis software using pgdspider 2.0.1.0^51^ and convert 1.3.1^52^.

### Genetic bottleneck analysis

Screens for signatures of genetic bottlenecks were carried out for cultivated and wild populations using bottleneck 1.2.02^31^. A one-tailed Wilcoxon signed-rank test was applied to determine the significance of heterozygosity excess, and 10,000 replicates were run under three microsatellite models: infinite allele model (IAM), stepwise-mutation model (SMM) and two-phase mutation model (TPM) incorporated with 90% IAM and 10% SMM^53^,^54^,^55^. A test for mode shift was also used to detect genetic bottlenecks. The expected distributions of allele frequencies exhibit a normal L-shaped distribution, but population bottlenecks could cause a characteristic mode-shift distortion in the distribution of allele frequencies^54^.

### Genetic differentiation analysis

Global and pairwise *F*_ST_ across all loci was investigated with correction for null alleles, using freena with 10,000 bootstrap resampling, to avoid the impact of null alleles on the estimation of genetic differentiation^44^. Pairwise genetic differentiation among the 28 populations was estimated over 100,000 random permutations with arlequin 3.5.1.3^56^.

Analysis of molecular variance (AMOVA) was conducted in arlequin 3.5.1.3 to partition genetic variation across nested levels: within populations, between populations within groups, and between groups^56^. The different groups were defined as follows (Table 1): the cultivated/introduced populations grouped 20 populations (NC + SC + WI); the wild indigenous populations grouped 8 populations (WR + WJ1 + WJ2); the northern cultivated populations (NC) included 12 populations; the southern cultivated populations (SC) possessed 5 populations.

### Analysis of population structure

To infer genetic clusters in *S. japonica* populations, multivariate analyses were carried out using discriminate analyses of principal components (DAPC) using the adegenet 1.3.1 package^57^,^58^ in the R environment (R Development Core Team 2013). The optimal number of clusters was selected based on the lowest Bayesian Information Criterion (BIC). Pairwise genetic distances between populations were calculated using Nei’s^59^ standardized genetic distance *Da*, and the non-rooted tree was generated using the neighbour-joining (NJ) algorithm with 1,000 bootstrap replicates in poptree^60^. Tree topologies were viewed and adjusted with figtree 1.4.2 (available at http://tree.bio.ed.ac.uk/software/figtree/). Bayesian analysis of the population genetic structure with structure 2.3.1 was applied with the admixture model and correlated allele frequencies^61^,^62^. To determine the optimal number of clusters, K, the population structure was tested at K values ranging from 1 to 15 with 20 replicates based on 1,000,000 Markov chain Monte Carlo iterations following a burn-in period of 500 000 steps, and we evaluated the log-likelihood [lnP(k)] for each K and estimated Delta (K) using structure harvester^63^. The graphical results were displayed using distruct^64^.

## Additional Information

**How to cite this article**: Zhang, J. *et al*. Effect of domestication on the genetic diversity and structure of *Saccharina japonica* populations in China. *Sci. Rep.* **7**, 42158; doi: 10.1038/srep42158 (2017).

**Publisher's note:** Springer Nature remains neutral with regard to jurisdictional claims in published maps and institutional affiliations.

## Supplementary Material

Supplementary Information

## Figures and Tables

**Table 1:** Genetic diversity analysis of 28 *Saccharina japonica* populations.

Population	N	*N*_A_	*A*_R_	*N*_P_	*H*_O_	*H*_E_	*F*_IS_
**Northern cultivated populations** (**NC**)							
ZK1	26	3.47 (0.31)	3.36 (0.29)	0.00 (0.00)	0.42 (0.04)	0.43 (0.04)	0.06
ZK2	27	3.32 (0.28)	3.15 (0.24)	0.00 (0.00)	0.44 (0.04)	0.43 (0.03)	−0.02
AL	24	2.84 (0.30)	2.77 (0.27)	0.00 (0.00)	0.44 (0.03)	0.45 (0.03)	0.03
901	26	2.53 (0.21)	2.51 (0.20)	0.00 (0.00)	0.37 (0.06)	0.33 (0.05)	−0.08*
DF2	26	1.95 (0.12)	1.91 (0.11)	0.00 (0.00)	0.78 (0.09)	0.40 (0.05)	−0.96**
DF3	21	3.68 (0.34)	3.66 (0.33)	0.00 (0.00)	0.60 (0.05)	0.55 (0.04)	−0.06
RF	30	2.68 (0.20)	2.51 (0.17)	0.00 (0.00)	0.30 (0.04)	0.32 (0.05)	0.07
TJ	30	3.16 (0.30)	2.95 (0.25)	0.00 (0.00)	0.40 (0.05)	0.38 (0.05)	−0.03
LJ	27	2.68 (0.17)	2.59 (0.16)	0.00 (0.00)	0.42 (0.04)	0.41 (0.04)	−0.01
NJ	28	3.00 (0.31)	2.86 (0.28)	0.00 (0.00)	0.40 (0.07)	0.38 (0.05)	−0.03
PL	28	3.74 (0.29)	3.54 (0.26)	0.00 (0.00)	0.47 (0.05)	0.43 (0.04)	−0.08**
YZ	25	2.84 (0.21)	2.80 (0.20)	0.00 (0.00)	0.43 (0.04)	0.42 (0.04)	0.00
Mean (SE)		2.99 (0.15)	2.88 (0.14)	0.00 (0.00)	0.46 (0.04)	0.41 (0.02)	
**Southern cultivated populations** (**SC**)							
HG	30	3.11 (0.25)	3.31 (0.25)	0.00 (0.00)	0.47 (0.04)	0.42 (0.03)	−0.11**
LZ	24	3.53 (0.28)	2.98 (0.21)	0.00 (0.00)	0.35 (0.05)	0.36 (0.04)	0.07
GW	21	2.00 (0.13)	1.99 (0.13)	0.00 (0.00)	0.30 (0.05)	0.28 (0.05)	−0.03
PT	33	2.16 (0.19)	2.06 (0.17)	0.00 (0.00)	0.35 (0.07)	0.30 (0.05)	−0.13**
XP	28	1.79 (0.10)	1.78 (0.10)	0.00 (0.00)	0.25 (0.05)	0.25 (0.04)	0.01
Mean (SE)		2.52 (0.34)	2.42 (0.30)	0.00 (0.00)	0.34 (0.04)	0.32 (0.03)	
**Wild introduced populations** (**WI**)							
XS	30	3.37 (0.29)	2.99 (0.23)	0.26 (0.13)	0.42 (0.05)	0.40 (0.04)	−0.02
YM	30	2.26 (0.24)	2.19 (0.21)	0.00 (0.00)	0.35 (0.05)	0.35 (0.05)	0.01
ZD	33	2.63 (0.28)	2.48 (0.25)	0.16 (0.09)	0.36 (0.05)	0.39 (0.04)	0.09*
Mean (SE)		2.75 (0.33)	2.55 (0.23)	0.14 (0.08)	0.38 (0.02)	0.38 (0.02)	
**Wild indigenous populations in Russia** (**WR**)							
EP	30	4.16 (0.65)	3.75 (0.59)	0.26 (0.13)	0.29 (0.07)	0.29 (0.06)	0.02
SH	28	6.00 (0.79)	5.33 (0.71)	0.32 (0.15)	0.48 (0.06)	0.49 (0.05)	0.04
AW	24	5.89 (1.31)	5.53 (1.19)	0.89 (0.52)	0.45 (0.06)	0.49 (0.06)	0.09**
Mean (SE)		5.35 (0.60)	4.87 (0.56)	0.49 (0.20)	0.41 (0.06)	0.42 (0.07)	
**Wild indigenous populations in Japan** (**WJ**)							
WA	27	5.05 (0.79)	4.69 (0.73)	0.16 (0.12)	0.46 (0.07)	0.47 (0.06)	0.04
SP	32	5.26 (0.75)	4.79 (0.67)	0.26 (0.10)	0.49 (0.06)	0.51 (0.06)	0.04
KA	22	6.79 (0.76)	6.64 (0.74)	0.21 (0.10)	0.61 (0.04)	0.63 (0.04)	0.05*
HA	28	9.00 (0.85)	8.23 (0.74)	0.89 (0.30)	0.68 (0.04)	0.71 (0.04)	0.06**
SA	28	9.11 (1.24)	8.19 (1.07)	0.89 (0.23)	0.64 (0.05)	0.68 (0.05)	0.07**
Mean (SE)		7.04 (0.88)	6.51 (0.78)	0.48 (0.17)	0.58 (0.04)	0.60 (0.05)	

N, numbers of individuals successfully genotyped; *N*a, mean number of alleles across loci. *A*_R_, allelic richness based on 20 samples per population; *N*_P_, number of private alleles; *H*_O_, observed heterozygosity averaged across loci; *H*_E_, expected heterozygosity across loci. *N*a, *A*_R_, *N*_P_, *H*_E_ and *H*_O_ are mean values over loci with standard errors in brackets. *F*_IS_, inbreeding coefficient calculated overall loci. ^*^P < 0.05; ^**^P < 0.01.

**Table 2:** Analysis of molecular variance (AMOVA) for different groups in *Saccharina japonica.*

Source of variation	Degree of freedom	Sum of squares	Variance of components	Percentage variation	*P* values
(a) *Cultivated/introduced populations vs. wild indigenous populations*					
Among groups	1	1486.64	2.27	30.49	*P* < 0.001
Among populations within groups	26	1660.41	1.09	14.67	*P* < 0.001
Within populations	1504	6150.88	4.09	54.85	
(b) *Northern cultivated populations vs. southern cultivated populations*					
Among groups	1	157.75	0.29	6.09	*P* < 0.001
Among populations within groups	15	682.16	0.78	16.41	*P* < 0.001
Within populations	891	3300.27	3.70	77.50	

(a) Global *Ф*_ST_ among populations without hierarchy is 0.45, *P* < 0.001; (b) Global *Ф*_ST_ among populations without hierarchy is 0.23, *P* < 0.001.
