# Supplementary material for: Effect of domestication on the genetic diversity and structure of Saccharina japonica populations in China
Source: Sci Rep. 2017 Feb 8;7:42158. doi: 10.1038/srep42158 (PMC5296902; doi:10.1038/srep42158)
Supplement: Supplementary Information [file srep42158-s1.pdf]

Effect of domestication on the genetic diversity and structure of *Saccharina japonica* populations in China

Jie Zhang[1,2,3], Xiuliang Wang[1,2], Jianting Yao[1,2,*], Qiuying Li[1,2,3], Fuli Liu[4], Norishige Yotsukura[5], Tatiana N Krupnova[6], Delin Duan[1,2,*]

Figure S1. Brief domestication history of *Saccharina japonica* in China[1-7].

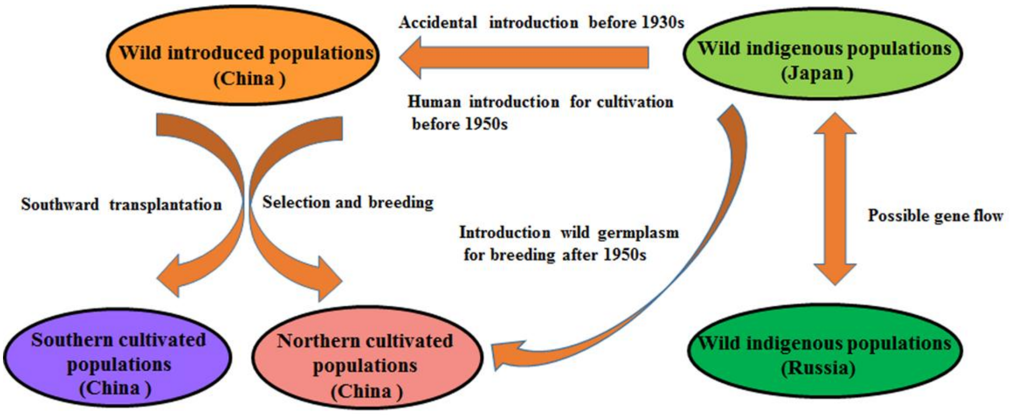

Figure S2. Determination of the optimal K values for *Saccharina japonica* populations implemented in STRUCTURE HARVESTER (http://taylor0.biology.ucla.edu/structureHarvester/) using the ΔK method and LnP(D). (a). Mean log probability of data LnP(D) over 20 replicates for each K values (error bars represent standard deviation); (b). ΔK over 20 replicates for each K values .

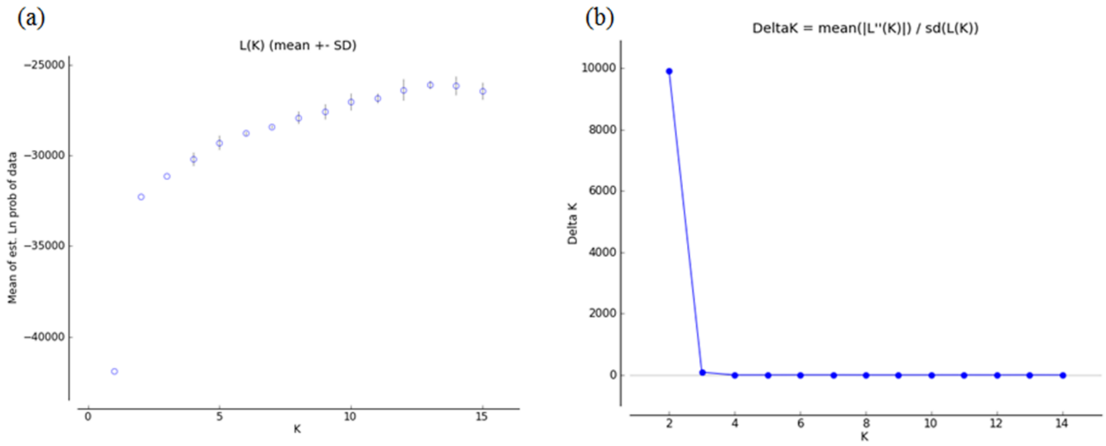

Table S1 Background of *Saccharina japonica* populations used in this study.

Code	Coordinate	Genetic background
**Northern cultivated populations (NC)**		
ZK1*	N 37°13′55″ E 122°35′54″	Zhongke No.1: descendants of *S. japonica* intraspecific hybridization[1]
ZK2*	N 37°13′39″ E 122°36′01″	Zhongke No.2: artificial selected from *S. japonica* aquaculture farm[1]
**AL***	N 37°13′25″ E 122°36′53″	Ailunwan (Benniu): inbreeding descendants of *S. japonica* landrace[1]
**901***	N 37°13′13″ E 122°36′04″	90-1: hybrid of *S. longissima* (maternal parent) and *S. japonica* (paternal parent)[2,3]
**DF2***	N 37°13′11″ E 122°36′12″	Dongfang No.2: F1 hybrids of *S. longissima* (paternal parent) and *S. japonica* (maternal parent)[4]
**DF3**	N 37°13′03″ E 122°36′40″	Dongfang No.3: F1 hybrids of *S. longissima* (maternal parent) and *S. japonica* (paternal parent)[5]
**RF**	N 37°13′50″ E 122°35′20″	Rongfu: hybrid of *S. japonica* and Yuanza NO.10 (*S. japonica* × *S. latissima*)[6]
TJ	N 38°09′18″ E 120°45′18″	Tuoji: artificial selected *S. japonica* from northern cultivars
LJ	N 37°09′39″ E 122°30′47″	Lijiang: artificial selected *S. japonica* from northern cultivars
NJ	N 36°55′20″ E 122°31′18″	Ningjin: artificial selected *S. japonica* from northern cultivars
PL	N 37°49′53″ E 120°46′16″	Penglai: artificial selected *S. japonica* from northern cultivars
YZ	N 36°45′47″ E 121°27′22″	Yanza: hybrid between *S. japonica* and *S. longissima*[7]
**Southern cultivated populations (SC)**		
**HG**	N 38°48′36″ E 121°23′24″	Huangguan No.1: descendants of *S. japonica* (southern cultivars) intraspecific hybridization[8]
LZ*	N 26°19′25″ E 119°49′02″	Lianza: artificial selected from descendants of transplanting *S. japonica* from northern China[1]
GW*	N 26°19′27″ E 119°49′17″	Guanwu: artificial selected from descendants of transplanting *S. japonica* from northern China[1]
PT	N 25°11′06″ E 119°14′42″	Putian: artificial selected from descendants of southern cultivars
XP	N 26°41′44″ E 119°55′34″	Xiapu: artificial selected from descendants of southern cultivars
**Wild invasive populations in China (WI)**		
XS*	N 37°09′54″ E 122°35′24″	*S. japonica* population grow in rocks of intertidal zone and have no clear origin information
YM*	N 37°29′24″ E 122°34′00″	*S. japonica* population grow in rocks of intertidal zone and have no clear origin information
ZD*	N 39°05′00″ E 122°47′00″	*S. japonica* population grow in rocks of intertidal zone and have no clear origin information
**Wild ingenious populations in Russia (WR)**		
EP*	N 43°06′54″ E 131°52′12″	Wild *S. japonica* in Eastern Primorsky
SH*	N 46°25′03″ E 141°51′0.7″	Wild *S. japonica* in Shebunino, Sakhalin
AW*	N 46°06′00″ E 143°18′00″	Wild *S. japonica* in western Aniva Bay, Sakhalin
**Wild ingenious populations in Japan (WJ)**		
WA*	N 45°27′01″ E 141°38′42″	Wild *S. japonica* in Wakkanai, Hokkaido
SP*	N 43°58′41″ E 144°52′32″	Wild *S. japonica* in Shiretoko Peninsula, Hokkaido
KA*	N 43°20′17″ E 140°23′27″	Wild *S. japonica* in Kamui cape, Hokkaido
HA*	N 41°46′00″ E 140°41′25″	Wild *S. japonica* in Hakodate, Hokkaido
SA	N 41°25′50″ E 141°27′37″	Wild *S. japonica* in Shiriya, Aomori pref, Honshu

Six cultivars (bold) have been examined and approved by the Chinese Approving Committee of Aquacultural Stock Seeds and Elite Varieties: “Huangguan No.1” (HG), “Rongfu” (RF), “Ailunwan” (AL), “90-1” (901), “Dongfang No.2” (DF2) and “Dongfang No.3” (DF3). The parentage information of other 11 cultivars was unclear, because most of these cultivars were hybrid and selected from different cultivated populations by local farmers and it is thus difficult to explore their genuine pedigree. Seven cultivated populations (*) had already been used in previous study[1]and ten wild invasive/indigenous populations (*) had already been used in previous study[9].

Reference:

1. Liu, F. L. *et al.* Genetic diversity and structure within and between wild and cultivated *Saccharina japonica* (Laminariales, Phaeophyta) revealed by SSR markers. *Aquaculture* **358-359**, 139-145, (2012).
2. Xia, P. *et al.* Genetic study of Kelp"901"strain. *Chin. J. Oceanol. Limn.* **23**, 152-157 (2005).
3. Zhang, Q. S. *et al.* Breeding of an elite *Laminaria* variety 90-1 through inter-specific gametophyte crossing. *J. Appl. Phycol.* **19**, 303-311 (2007).
4. Li, X. J. *et al.* Trait evaluation and trial cultivation of Dongfang No. 2, the hybrid of a male gametophyte clone of *Laminaria longissima* (Laminariales, Phaeophyta) and a female one of *L. japonica*. *J. Appl. Phycol.* **19**, 139-151, (2007).
5. Li, X. J. *et al.* Breeding and trial cultivation of Dongfang No. 3, a hybrid of *Laminaria* gametophyte clones with a more than intraspecific but less than interspecific relationship. *Aquaculture* **280**, 76-80 (2008).
6. Zhang, J. *et al.* Study on high-temperature-resistant and high-yield *Laminaria* variety “Rongfu”. *J. Appl. Phycol.* **23**, 165-171 (2011).
7. Shan, T. F., Pang, S. J., Zhang, Y. R., Yakovleva, I. M. & Skriptsova, A. V. An AFLP-based survey of genetic diversity and relationships of major farmed cultivars and geographically isolated wild populations of *Saccharina japonica* (Phaeophyta) along the northwest coasts of the Pacific. *J. Appl. Phycol.* **23**, 35-45 (2010).
8. Liu, F. L. *et al.* Breeding, economic traits evaluation, and commercial cultivation of a new *Saccharina* variety “Huangguan No. 1”. *Aquac. Int.* **22**, 1665-1675 (2014).
9. Zhang, J. *et al.* Phylogeographic data revealed shallow genetic structure in the kelp Saccharina japonica (Laminariales, Phaeophyta). *BMC Evol. Biol.* **15**, 237 (2015)

Table S2. Characteristic parameters of 23 SSR loci detected in *Saccharina japonica* populations

SSR	P_A (%)	A	PIC	H_E	H_O	NULL
SJ3	99.6	14	0.59	0.42 (0.05)	0.38 (0.04)	0.00 (0.00)
SJ13	100	9	0.60	0.24 (0.05)	0.36 (0.04)	0.10 (0.02)
SJ80	99.6	20	0.63	0.44 (0.06)	0.41 (0.05)	0.01 (0.00)
SJ20	100	15	0.72	0.50 (0.04)	0.49 (0.03)	0.02 (0.01)
SJ66	99.9	11	0.55	0.37 (0.04)	0.39 (0.04)	0.03 (0.01)
SJ95	99.9	30	0.77	0.55 (0.04)	0.56 (0.04)	0.03 (0.01)
SJ133	100	10	0.35	0.31 (0.05)	0.29 (0.04)	0.02 (0.01)
SJ106	100	11	0.65	0.42 (0.04)	0.41 (0.03)	0.03 (0.01)
SJ132	99.7	36	0.83	0.72 (0.04)	0.68 (0.04)	0.02 (0.01)
SJ52	100	9	0.65	0.43 (0.05)	0.41 (0.04)	0.02 (0.01)
SJ31	100	9	0.22	0.18 (0.05)	0.16 (0.04)	0.01 (0.01)
SJ93	99.7	7	0.50	0.28 (0.05)	0.28 (0.04)	0.03 (0.01)
SJ86	100	14	0.56	0.44 (0.04)	0.46 (0.03)	0.03 (0.01)
SJ102	99.6	14	0.69	0.52 (0.05)	0.50 (0.04)	0.02 (0.01)
SJ99	100	22	0.52	0.45 (0.04)	0.45 (0.03)	0.04 (0.01)
SJ110	99.9	20	0.76	0.59 (0.05)	0.56 (0.04)	0.02 (0.01)
SJ101	100	12	0.56	0.37 (0.04)	0.39 (0.04)	0.03 (0.01)
SJ21	99.9	14	0.67	0.36 (0.05)	0.43 (0.04)	0.07 (0.02)
SJ113	99.5	16	0.65	0.42 (0.04)	0.43 (0.04)	0.04 (0.01)
SJ125	96.0	14	0.63	0.19 (0.03)	0.39 (0.06)	0.13 (0.02)
SJ24	99.9	19	0.73	0.62 (0.04)	0.61 (0.03)	0.02 (0.01)
SJ114	99.9	9	0.57	0.42 (0.04)	0.40 (0.03)	0.02 (0.01)
SJ136	100	50	0.78	0.36 (0.04)	0.61 (0.04)	0.16 (0.02)

P_A , percentage of successful amplification per locus; A, alleles number; PIC, polymorphic information content; H_E, mean expected heterozygosity across populations with standard error (SE); H_O, mean observed heterozygosity across populations with standard error (SE); NULL, mean frequency of null alleles across populations with standard error (SE).

Table S3 Genetic diversity (H_S), inbreeding coefficient (F_{IS}) and pairwise genetic differentiation F_{ST} values were calculated among populations within one group with FSTAT software. These indices were compared between groups with the same software using a permutation procedure: the two-tailed P values were obtained after 1000 permutations.

Group	Genetic diversity (H_S)	F_{IS}	F_{ST}
Cultivated populations (NC and SC)	0.390	-0.078	0.199
Wild indigenous populations (WR and WJ)	0.539	0.054	0.247
P values	0.003	0.06	0.591
Northern cultivated populations (NC)	0.415	-0.097	0.192
Southern cultivated populations (SC)	0.328	-0.019	0.145
Wild introduced populations (WI)	0.386	0.030	0.063
Wild indigenous populations in Russia (WR)	0.426	0.053	0.305
Wild indigenous populations in Japan (WJ)	0.607	0.054	0.158
P values	0.010	0.542	0.636

Table S4 Bottleneck tests of heterozygosity excess in 28 *Saccharina japonica* populations. Tests of heterozygosity excess were performed under three microsatellite mutation models (I.A.M: infinite allele model; S.M.M: stepwise mutation; T.P.M: two-phase mutation model), and all were assumed in mutation-drift equilibrium. *P* values for heterozygosity excess after FDR correction were in brackets, and significant *P* value (< 0.05) were indicated in bold. The allele frequency distribution was tested against a null hypothesis of exponential decline (L-shape; Shifted mode)

**Code**	**Under the I.A.M**	**Under the S.M.M**	**Under the T.P.M**	**Mode-shift**
**Northern cultivated populations (NC)**				
**ZK1**	0.05890	0.96673	0.56748	L-shaped distribution
**ZK2**	0.10455	0.92180	0.53914	L-shaped distribution
**AL**	**0.00357 (0.014)**	0.20902	0.01291	L-shaped distribution
**901**	0.06010	0.69439	0.32207	L-shaped distribution
**DF2**	**0.00004 (0.001)**	**0.00004 (0.001)**	**0.00004 (0.001)**	**Shifted mode**
**DF3**	**0.00005 (0.001)**	0.24754	0.00602	L-shaped distribution
**RF**	0.28992	0.93513	0.72456	L-shaped distribution
**TJ**	0.18461	0.85814	0.56748	L-shaped distribution
**LJ**	**0.00473 (0.017)**	0.42979	0.04776	L-shaped distribution
**NJ**	0.07702	0.75246	0.38301	L-shaped distribution
**PL**	0.27061	0.99987	0.96686	L-shaped distribution
**YZ**	**0.00142 (0.013)**	0.39913	0.05669	L-shaped distribution
**Southern cultivated populations (SC)**				
**HG**	**0.00229 (0.013)**	0.88734	0.23266	L-shaped distribution
**LZ**	0.82673	0.99974	0.98482	L-shaped distribution
**GW**	0.02533	0.21660	0.11560	L-shaped distribution
**PT**	**0.00903 (0.028)**	0.17957	0.06027	L-shaped distribution
**XP**	**0.01077 (0.030)**	0.06769	0.03186	**Shifted mode**
**Wild invasive populations (WI)**				
**XS**	0.15190	0.99088	0.71008	L-shaped distribution
**YM**	**0.00278 (0.013)**	0.05444	0.01161	L-shaped distribution
**ZD**	**0.00167 (0.012)**	0.17623	0.01617	L-shaped distribution
**Wild indigenous populations in Russia (WR)**				
**EP**	0.98839	0.99990	0.99999	L-shaped distribution
**SH**	0.82377	0.99915	0.98553	L-shaped distribution
**AW**	0.51839	0.99966	0.97156	L-shaped distribution
**Wild indigenous populations in Japan (WJ)**				
**WA**	0.38301	0.98658	0.83764	L-shaped distribution
**SP**	0.09073	0.99832	0.79144	L-shaped distribution
**KA**	0.15620	0.99833	0.86233	L-shaped distribution
**HA**	0.15620	0.99941	0.96990	L-shaped distribution
**SA**	0.09782	0.99915	0.83397	L-shaped distribution

Table S5 Pairwise values of F_{ST} (below diagonal) and *P*-values (above) after FDR correction between 28 *Saccharina japonica* populations for 16 SSR loci. Bold indicated non-significant *P*-values ($P > 0.05$). F_{ST} values between Chinese populations and wild indigenous populations are in shaded.

	ZK1	ZK2	AL	901	DF2	DF3	RF	TJ	LJ	NJ	PL	YZ	HG	LZ	GW	PT	XP	XS	YM	ZD	EP	SH	AW	WA	SP	KA	HA	SA
**ZK1**		NS	***	***	***	***	***	***	*	*	***	***	***	***	***	***	***	***	***	***	***	***	***	***	***	***	***	***
**ZK2**	**0.009**		***	***	***	***	***	***	***	*	***	***	***	***	***	***	***	***	***	***	***	***	***	***	***	***	***	***
**AL**	0.056	0.041		***	***	***	***	***	NS	***	***	***	***	***	***	***	***	***	***	***	***	***	***	***	***	***	***	***
**901**	0.123	0.083	0.142		***	***	*	***	***	***	***	***	***	***	***	***	***	***	***	***	***	***	***	***	***	***	***	***
**DF2**	0.271	0.272	0.224	0.368		***	***	***	***	***	***	***	***	***	***	***	***	***	***	***	***	***	***	***	***	***	***	***
**DF3**	0.356	0.367	0.364	0.418	0.426		***	***	***	***	***	***	***	***	***	***	***	***	***	***	***	***	***	***	***	***	***	***
**RF**	0.105	0.076	0.137	0.025	0.395	0.433		***	***	***	***	***	***	***	***	***	***	***	***	***	***	***	***	***	***	***	***	***
**TJ**	0.018	0.015	0.056	0.137	0.317	0.392	0.123		***	***	**	**	***	***	***	***	***	***	***	***	***	***	***	***	***	***	***	***
**LJ**	0.068	0.058	0.009	0.188	0.265	0.394	0.173	0.051		***	***	***	***	***	***	***	***	***	***	***	***	***	***	***	***	***	***	***
**NJ**	0.140	0.123	**0.173**	0.238	0.346	0.378	0.250	0.118	0.170		***	***	***	***	***	***	***	***	***	***	***	***	***	***	***	***	***	***
**PL**	0.043	0.024	0.063	0.135	0.293	0.368	0.131	0.023	0.059	0.098		**	***	***	***	***	***	***	***	***	***	***	***	***	***	***	***	***
**YZ**	0.047	0.035	0.050	0.136	0.278	0.379	0.133	0.040	0.039	0.159	0.022		***	***	***	***	***	***	***	***	***	***	***	***	***	***	***	***
**HG**	0.070	0.112	0.181	0.261	0.367	0.378	0.218	0.138	0.189	0.224	0.149	0.159		***	***	***	***	***	***	***	***	***	***	***	***	***	***	***
**LZ**	0.019	0.037	0.059	0.174	0.283	0.376	0.140	0.052	0.060	0.170	0.062	0.062	0.064		NS	***	***	***	***	***	***	***	***	***	***	***	***	***
**GW**	0.071	0.121	0.186	0.289	0.394	0.442	0.231	0.138	0.187	0.263	0.166	0.170	0.004	**0.064**		**	***	***	***	***	***	***	***	***	***	***	***	***
**PT**	0.102	0.146	0.215	0.288	0.440	0.458	0.225	0.160	0.215	0.308	0.196	0.192	0.059	0.109	0.041		***	***	***	***	***	***	***	***	***	***	***	***
**XP**	0.157	0.173	0.229	0.330	0.399	0.486	0.295	0.170	0.188	0.252	0.156	0.148	0.173	0.131	0.177	0.230		***	***	***	***	***	***	***	***	***	***	***
**XS**	0.109	0.117	0.134	0.238	0.302	0.393	0.214	0.136	0.149	0.184	0.111	0.123	0.122	0.101	0.136	0.161	0.168		***	***	***	***	***	***	***	***	***	***
**YM**	0.106	0.138	0.175	0.249	0.357	0.436	0.231	0.174	0.205	0.239	0.161	0.175	0.152	0.113	0.169	0.210	0.276	0.088		***	***	***	***	***	***	***	***	***
**ZD**	0.075	0.100	0.138	0.227	0.315	0.407	0.206	0.117	0.153	0.196	0.117	0.124	0.093	0.069	0.096	0.138	0.149	0.037	0.069		***	***	***	***	***	***	***	***
**EP**	0.581	0.588	0.578	0.637	0.602	0.472	0.650	0.609	0.599	0.602	0.581	0.586	0.627	0.596	0.672	0.660	0.695	0.600	0.633	0.616		***	***	***	***	***	***	***
**SH**	0.459	0.470	0.463	0.517	0.470	0.340	0.535	0.497	0.487	0.485	0.466	0.462	0.504	0.485	0.547	0.551	0.582	0.478	0.514	0.502	0.256		***	***	***	***	***	***
**AW**	0.453	0.461	0.449	0.512	0.447	0.322	0.531	0.489	0.478	0.473	0.459	0.453	0.496	0.474	0.539	0.546	0.575	0.456	0.505	0.487	0.457	0.175		***	***	***	***	***
**WA**	0.439	0.447	0.444	0.503	0.453	0.361	0.522	0.470	0.464	0.456	0.439	0.449	0.494	0.460	0.534	0.543	0.561	0.457	0.502	0.480	0.459	0.335	0.319		***	***	***	***
**SP**	0.408	0.412	0.412	0.470	0.408	0.325	0.490	0.435	0.433	0.404	0.399	0.410	0.452	0.426	0.494	0.509	0.518	0.413	0.460	0.439	0.434	0.301	0.290	0.068		***	***	***
**KA**	0.340	0.345	0.344	0.394	0.368	0.240	0.409	0.379	0.377	0.360	0.340	0.356	0.375	0.357	0.425	0.439	0.465	0.353	0.398	0.380	0.386	0.230	0.248	0.210	0.199		***	***
**HA**	0.283	0.297	0.277	0.342	0.301	0.152	0.366	0.316	0.311	0.304	0.289	0.288	0.323	0.308	0.364	0.389	0.403	0.307	0.342	0.327	0.325	0.176	0.179	0.223	0.184	0.115		***
**SA**	0.294	0.306	0.297	0.351	0.310	0.158	0.376	0.330	0.332	0.307	0.303	0.307	0.328	0.325	0.375	0.399	0.419	0.322	0.362	0.344	0.329	0.189	0.190	0.238	0.184	0.120	0.029	

* $P < 0.05$; ** $P < 0.001$; *** $P < 0.0001$; NS, not significant.

Table S6 The primer information of SSR markers (Li *et al.*, 2015)

**No**	**SSR**	**Motif**	**Forward primer (5'-3')**	**Reverse primer (5'-3')**	**Tm (℃)**	**size (bp)**
	SJ3	(CTG)10	CTTATTCGTGCTGCGTTCATAC	AGTTTGAGCCTCTGCGTCAT		321
1	SJ13	(GTCGG)7	CAGCAGATGGCGACTTACTTTAG	GTCTTGACGGAGCGTTTGAT	55	279
	SJ80	(GCA)10	CTCCGACTGCCATACTCTTTGAC	CTTCGTGCTTACGTATATCCGAGTT		321
	SJ20	(TTGCT)7	AGAGAACGCGGAGTGGAACT	CGCACTCGTCACAACGTGTAT		299
2	SJ66	(CCATA)5	GACTCCTCCGACTCGATCCT	ATAAGTTCTCCCGAAGCTGTTTCT	58	296
	SJ95	(TCGG)5	CGGTTAGGCACTCCATTGTATC	GCCAGTCGAAACTCAATACTCATAC		327
	SJ133	(CAG)8	AGCGATAATCAGGAAATCGTCTC	GTTTGGGTTGTTCTGTCACATTC		319
3	SJ106	(TGT)7	ACAAGAAGAGCGCACAGAGAAT	ATACGTACGTGATAGCTGGACTTC	58	281
	SJ132	(TAG)11	GGTATACTTTCTCCGCCATCAAG	GCACCGAGTTTCTACACCTGTT		329
	SJ52	(AGGC)5	ATGAAGGCAATCATCATCAGGT	TAGCACCAACGCTCTAGAATAGAAG		298
4	SJ31	(CAG)6	AAGCAGCAGCCATCATGTTAC	CTGGTCAAGTCTCTGCTCTCATC	57	267
	SJ93	(AAAC)7	CACCCTTATCATCCCTGTTCAAG	TTCGGGACAAGAGTGATACATAGTT		316
	SJ86	(TTAT)6	AGTAGAAGCGGCAGTAGGTAGGAC	AAGAGCAACACATACACATGCATAC		313
5	SJ102	(TGC)6	TACTGTACTGCAGTGCTTGCGATTG	CAGCAGCACTACTCCTAGGTACATT	57	274
	SJ99	(ACAG)5	AAAGGTTGGACAGACACGATATG	ATCCCTTTCTCTTTCTCGTTCTCT		321
6	SJ110	(GTA)7	GGACGAAGTTATGGACTCGTTTC	GTATCACCCGTAGTTTCCTCACC	57	291
	SJ101	(CGTA)8	ACAACGCTCCCAAGAGACTG	GGTCGCATGGTAAGCTCTTCT		326
	SJ21	(CCTTC)7	TTTGTCCGCTCTCGCTCTT	GATGTCCTTGCCCTTGAAATAC		309
7	SJ113	(TGC)9	GAACGAACGGACGAATCGTAT	GTGGACCACAGGGAGAGAGAT	55	281
	SJ125	(GAAG)7	CCTCAGCTTCTCGATCCTCTT	CTTTGGTTGTCGTTGTTGTTATCC		327
	SJ24	(GAACC)7	CACGTCTGCTTCTGCTCTACAAC	CGTAGATGATGAACAGGCTGAAC		306
8	SJ114	(CAC)9	AGCAGCAGAGCTGGAGACTG	GTCGTGTCCGAGTAGGAGGTTAG	57	282
	SJ136	(TGTCC)7	GGGTATCTCTCGGAATGATAGAAC	AGAGCTAGCAGTAGCTGTGGAAAT		329

Reference:

Li, Q. Y., Zhang, J., Yao, J. T., Wang, X. L. & Duan, D. L. Development of *Saccharina japonica* genomic SSR markers using next-generation sequencing. *J. Appl. Phycol.* doi:10.1007/s10811-015-0643-0 (2015).
